# Predicting survival outcomes in dedifferentiated chondrosarcoma: a prognostic factor analysis from a National Registry

**DOI:** 10.1051/sicotj/2025011

**Published:** 2025-03-13

**Authors:** Tomoya Masunaga, Shinji Tsukamoto, Kanya Honoki, Hiromasa Fujii, Akira Kido, Manabu Akahane, Yasuhito Tanaka, Andreas F. Mavrogenis, Costantino Errani, Akira Kawai

**Affiliations:** 1 Department of Orthopaedic Surgery, Nara Medical University 840, Shijo-cho Kashihara-city Nara 634-8521 Japan; 2 Department of Rehabilitation Medicine, Nara Medical University 840, Shijo-cho Kashihara-city Nara 634-8521 Japan; 3 Department of Health and Welfare Services, National Institute of Public Health 2-3-6 Minami Wako-shi, Saitama 351-0197 Japan; 4 First Department of Orthopaedics, National and Kapodistrian University of Athens, School of Medicine 41 Ventouri Street 15562 Holargos Athens Greece; 5 Department of Orthopaedic Oncology, IRCCS Istituto Ortopedico Rizzoli Via Pupilli 1 40136 Bologna Italy; 6 Division of Musculoskeletal Oncology, National Cancer Center Hospital 5-1-1 Tsukiji, Chuoku Tokyo 104-0045 Japan

**Keywords:** Dedifferentiated chondrosarcoma, Adjuvant, Chemotherapy, Surgery, Radiotherapy

## Abstract

*Introduction*: Dedifferentiated chondrosarcoma (DDCS) is a high-grade subtype of chondrosarcoma with a poor prognosis. Treatment for localized DDCS generally involves wide resection; the effectiveness of adjuvant radiotherapy and chemotherapy is questionable. This research was designed to find prognostic factors for DDCS and evaluate the impact of adjuvant therapies on localized cases. *Methods*: One hundred thirty-two patients with DDCS diagnosed by pathology in the period 2006 to 2022 were identified in the Japanese National Bone and Soft Tissue Tumor Registry database and were retrospectively analyzed. *Results*: Patients with distant metastases at diagnosis (*n* = 34) had significantly poorer survival than those without metastases (*n* = 98), with a 5-year disease-specific survival (DSS) of 9.7% vs. 37.1% (*P* < 0.0001). For patients without distant metastasis at diagnosis, uni- and multivariate analysis showed that R1 or R2 surgical margin was an independent risk factor linked with unfavorable local recurrence (hazard ratio [HR] 3.39 [95% CI: 1.35–8.52]; *P* = 0.010). Adjuvant radiotherapy was not associated with local recurrence (HR 2.41 [95% CI: 0.87–6.64]; *P* = 0.090). Larger size (HR 1.13 [95% CI: 1.06–1.19]; *P* < 0.001) and no surgery (HR 3.87 [95% CI: 1.61–9.28]; *P* = 0.002) were independent risk factors for unfavorable DSS. Previous surgery (HR 0.19 [95% CI: 0.04–0.84]; *P* = 0.028) and adjuvant chemotherapy (HR 0.36 [95% CI: 0.16–0.77]; *P* = 0.009) were independent risk factors for favorable DSS. *Discussion*: Survival may have been improved by chemotherapy, but the effect of adjuvant radiotherapy in controlling the local spread of the tumor appears to have been limited in DDCS cases that were localized.

## Introduction

Dedifferentiated chondrosarcoma (DDCS) is a high-grade chondrosarcoma subtype that is histologically biomorphic, consisting of conventional chondrosarcoma that abruptly morphs into a non-cartilaginous, high-grade sarcoma [1]. DDCS makes up 6 to 10% of all chondrosarcomas and approximately 2% of primary malignant bone tumors [2]. Depending on where the tumor is located, there are considered to be two different radiographic subtypes of DDCS: (1) central, arising from an enchondroma in the medullary canal, and (2) peripheral, the origin of which is an osteochondroma of the bone cortex [1]; these are genetically distinct [3]. A proportion of central chondrosarcomas (approximately 15%) [4] and of peripheral chondrosarcomas (around 6%) [5] undergoes dedifferentiation. Lung metastases frequently occur, resulting in a 5-year survival rate of between 10 and 24% [4, 6–8].

Treatment of localized DDCS is normally by wide resection, and whether adjuvant chemotherapy should be administered is contested, as it reportedly improved prognosis in some cases [5, 7, 9] but not others [4, 6, 10–12]. In one prospective single-arm study, 57 patients who underwent surgery together with adjuvant chemotherapy showed an estimated 5-year overall survival (OS) of 39% [13], superior to the rate reported in previous retrospective studies (range 10–24%) [4, 6–8]. The recommendations of the National Comprehensive Cancer Network state that localized DDCS be treated according to the osteosarcoma protocol by wide resection plus adjuvant chemotherapy [14]. Guidelines of the European Society for Medical Oncology recommend that consideration also be given to adjuvant chemotherapy in cases of localized DDCS [15]. However, because DDCS only rarely occurs, only retrospective studies have been carried out, apart from one prospective study which lacked a control [13], and the effectiveness of adjuvant chemotherapy has not been studied in randomized controlled trials, so the efficacy of the latter in cases of localized DDCS is not clear.

Although chondrosarcomas are generally considered resistant to radiation, radiotherapy is sometimes employed as an adjuvant treatment with the aim of achieving local control. Gao et al. examined the data (*n* = 157) in the Surveillance, Epidemiology, and End Results (SEER) database from 1973 to 2014 and reported a possible improvement in OS with adjuvant radiotherapy (*P* = 0.015) [16], but there have not been any other coherent studies into the effectiveness of radiotherapy as an adjuvant treatment for localized DDCS. Thus its efficacy remains controversial.

We investigated prognostic factors in DDCS, focusing on patient outcomes after adjuvant radiotherapy and chemotherapy for cases of localized DDCS.

## Materials and methods

Using the database of the Japanese National Bone and Soft Tissue Tumor Registry [2], we selected 141 patients with DDCS who had been diagnosed by histopathological examination between January 2006 and December 2022. Nine patients were excluded due to unknown tumor size, and the remaining 132 patients were analyzed retrospectively.

Using these records, the following data were obtained: sex, age, previous surgery, tumor site, tumor size, distant metastasis at presentation, biopsy method, surgical margin [microscopically-negative margin defined as R0; macroscopically-negative but microscopically-positive margin as R1, and macroscopically-positive margin as R2] [17], adjuvant radiotherapy (target dose 60 Gy) [18], adjuvant chemotherapy, local recurrence, distant metastasis, and disease-specific survival (DSS). Age was categorized into the following groups: 0–39 years (children, adolescents, and young adults), 40–64 years (adults), and ≥ 65 years (elderly). The tumors were also grouped according to location, whether they occurred in the trunk or the upper or lower extremities. Tumor size was classified based on the 8th edition of the American Joint Committee on Cancer Staging Manual, in which bone tumors are classified by size (≤ 8 cm: T1 lesions, > 8 cm: T2 lesions) [19].

To evaluate any association between two variables, we used either the chi-square test or Fisher’s exact test, while the Mann‐Whitney U test was used for nonparametric statistical analysis of the difference between two independent samples. We defined local recurrence-free survival as the length of time between surgery and local recurrence or final follow-up, distant metastasis-free survival was defined as the time interval between initial diagnosis and distant metastasis or final follow-up, and DSS as the time period between diagnosis and death as a result of the disease, or from the initial diagnosis to final follow-up. The correlation between each of these variables and the different survival times was examined by univariate analysis using Cox proportional hazards regression analysis and Kaplan-Meier survival analysis (a log-rank test). Stepwise Cox proportional hazards regression, including all variables, was used for multivariate analysis. Statistical significance was set at *P* < 0.05. Analyses were performed using JMP 17 (SAS Institute Inc., Cary, NC, USA) and IBM SPSS (version 28.0; IBM Co., Armonk, NY, USA).

## Results

The 132 patients comprised 79 (59.8%) males and 53 (40.2%) females, and the median age of the study population was 68 (interquartile range [IQR], 56.3–75). Twelve (9.1%) had undergone previous surgery. The tumors were cited in the trunk in 58 patients (seven sternum, two scapula, two ischium, ten pubis, 21 ilium, 15 rib, one cervical vertebra), upper extremities in 24 patients (one phalanx, two metacarpals, 20 proximal humerus, one humeral diaphysis), and lower extremities in 50 patients (29 proximal femur, six femoral diaphysis, 14 distal femur, one distal tibia); the median diameter of the tumor was 10 cm (IQR, 8–14). Of the 132 patients, 98 had no distant metastases when they were first diagnosed, but the other 34 were found to have distant metastases. One patient had multiple hereditary exostoses, and one had Ollier’s disease. DSS was 56.1% at 1 year, 44.8% at 2 years, and 30.4% at 5 years, and the median DSS was 11 months (IQR, 3.3–33.5).

### Patients with metastases at diagnosis

Thirty-four patients were diagnosed when they already had distant metastases, located in the lung in 23 patients, bone in six, adrenal gland in two, brain in one, and two had pleural dissemination. Median patient age was 65 (IQR, 53.3–73), and five patients (14.7%) had undergone previous surgery. Sixteen patients had tumors in the trunk (one sternum, one scapula, two pubis, four ilium, eight rib), five in the upper extremities (five proximal humerus), and 13 in the lower extremities (eight proximal femur, two femoral diaphysis, and three distal femur). The median tumor diameter was 11.3 cm (IQR, 8.4–14.4). The diagnosis was the result of a needle biopsy in five patients (14.7%), an incisional biopsy in 26 (76.5%), and in three patients (8.8%) no biopsy was performed.

In regard to surgery, 16 patients were treated by R0 resection, two by R1 resection, and one by R2 resection; 15 had no surgery. Fourteen patients received chemotherapy for advanced disease, nine received adriamycin and ifosfamide regimens, three received adriamycin monotherapy, one received gemcitabine and docetaxel regimens, and two received pazopanib. Five patients received palliative radiotherapy.

**Figure 1:**
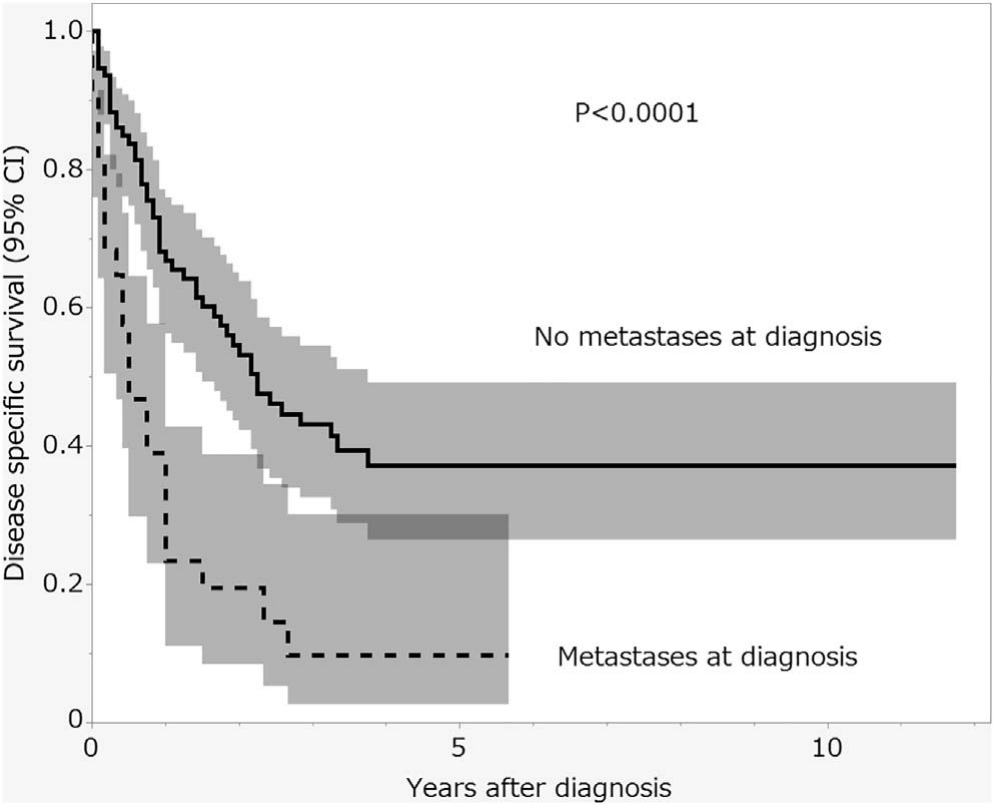
Comparison of the rates of disease-specific survival in patients who were found to have distant metastases at diagnosis, and in those who did not. The shaded areas around the curves indicates the 95% confidence interval.

DSS was 23.4% at 1 year, 19.5% at 2 years, and 9.7% at 5 years, making the median DSS 5 months (IQR, 1–12). Univariate analysis revealed that survival was significantly shorter in the patients who already had distant metastases at diagnosis compared to those without (5-year DSS 9.7% [95% confidence interval (CI): 2.6–30.1] vs. 37.1% [95% CI: 26.5–49.2]; *P* < 0.0001; Figure 1).

### Patients without metastases at diagnosis

Ninety-eight patients were found not to have any distant metastases when they were diagnosed. Regarding surgery, 70 patients were treated by R0 resection, six by R1 resection, and eight by R2 resection. Fourteen had no surgery. Local treatment was classified into three categories: R0 surgical margin, R1 or R2, and no surgery. Of the 14 patients who were not treated with surgery, three patients received carbon ion radiotherapy; two received proton beam therapy, and one received intensity-modulated radiation therapy.

The median age was 69 (IQR, 58–75), and tumor location was the trunk in 42 patients (six sternum, one scapula, two ischium, eight pubis, 17 ilium, seven rib, one cervical vertebra), upper limb in 19 (one phalanx, two metacarpals, 15 proximal humerus, one humeral diaphysis), and lower limb in 37 (21 proximal femur, four femoral diaphysis, 12 distal femur). Median tumor size was 10 cm (IQR, 8–13.3).

Twenty patients received adjuvant chemotherapy, eight received an adriamycin and ifosfamide regimen, and 12 received adriamycin and cisplatin. Thirteen patients received advanced chemotherapy: six received an adriamycin and ifosfamide regimen, six received an adriamycin and cisplatin regimen, two received adriamycin monotherapy, two received gemcitabine and docetaxel regimens, and one patient with high microsatellite instability received pembrolizumab. As shown in Table 1, adjuvant chemotherapy was more frequently administered to younger patients (*P* = 0.010). The follow-up period was longer in those given adjuvant chemotherapy than in those without (*P* = 0.023). Regarding other factors, no correlation was observed with the presence or absence of adjuvant chemotherapy.

**Table 1:** Association of adjuvant chemotherapy with clinical variables in the patients with localized dedifferentiated chondrosarcoma.

		Adjuvant chemotherapy		
Variable	No. of patients (*n* = 98)	No (*n* = 78)	Yes (*n* = 20)	*P* value
Age (years), *n* (%)				0.019*
< 40	2 (2.0)	1 (1.3)	1 (5.0)	
40–65	40 (40.8)	27 (34.6)	13 (65.0)	
> 65	56 (57.1)	50 (64.1)	6 (30.0)	
Sex, *n* (%)				0.776
Male	61 (62.2)	48 (61.5)	13 (65.0)	
Female	37 (37.8)	30 (38.5)	7 (35.0)	
Previous surgery, *n* (%)				0.339^#^
No	91 (92.9)	71 (91.0)	20 (100)	
Yes	7 (7.1)	7 (9.0)	0 (0)	
Site, *n* (%)				0.156
Trunk	41 (41.8)	29 (37.2)	12 (60.0)	
Upper limb	20 (20.4)	18 (23.1)	2 (10.0)	
Lower limb	37 (37.8)	31 (39.7)	6 (30.0)	
Tumor size (cm), *n* (%)				0.145^#^
< 8	23 (23.5)	21 (26.9)	2 (10.0)	
≥ 8	75 (76.5)	57 (73.1)	18 (90.0)	
Biopsy, *n* (%)				0.274
None	10 (10.2)	9 (11.5)	1 (5.0)	
Needle	6 (6.1)	6 (7.7)	0	
Open	82 (83.7)	63 (80.8)	19 (95.0)	
Surgical margin, *n* (%)				0.988
R0	70 (71.4)	56 (71.8)	14 (70.0)	
R1 or R2	14 (14.3)	11 (14.1)	3 (15.0)	
No surgery	14 (14.3)	11 (14.1)	3 (15.0)	
Adjuvant radiotherapy, *n* (%)				0.113^#^
No	87 (88.8)	67 (85.9)	20 (100)	
Yes	11 (11.2)	11 (14.1)	0	
Local recurrence, *n* (%)				0.602^#^
No	67 (77.0)	53 (76.8)	14 (77.8)	
Yes	20 (23.0)	16 (23.2)	4 (22.2)	
Distant metastases, *n* (%)				0.361
No	45 (45.9)	34 (43.6)	11 (55.0)	
Yes	53 (54.1)	44 (56.4)	9 (45.0)	
Died of disease, *n* (%)				0.316
No	49 (50.0)	37 (47.4)	12 (60.0)	
Yes	49 (50.0)	41 (52.6)	8 (40.0)	
Follow-up period after diagnosis (months), median (IQR)	14.5 (5.8–36.8)	11 (4–34.6)	25 (11.5–59.8)	0.023*

* Statistically significant.

# Fisher exact test was used.

Eleven patients received adjuvant radiotherapy. The total dose in all cases was 50–66 Gy, administered postoperatively in 1.8–2 Gy fractions, and no patient received preoperative irradiation. Eight patients received palliative radiotherapy.

Twenty patients experienced local recurrence (23.0%), with a median time period between excision surgery and diagnosis of local recurrence of 6.5 months (IQR, 3–9). Distant metastases were found in 53 patients (54.1%), with a median interval between the first diagnosis and the incidence of distant metastasis of 4.0 months (IQR, 2–11). Forty-nine patients (50%) died from their tumor, and the median DSS and follow-up period were both 14.5 months (IQR, 5.8–36.8).

To investigate cases of local recurrence, we excluded the 14 patients who were not treated by surgery, and data from the remaining 84 patients were used for univariate and multivariate analyses. Results showed that R1 or R2 surgical margins were an independent risk factor linked with unfavorable local recurrence (hazard ratio [HR] 3.39 [95% CI: 1.35–8.52]; *P* = 0.010) (Table 2). Adjuvant radiotherapy was not correlated with local recurrence rate (HR 2.41 [95% CI: 0.87–6.64]; *P* = 0.090) (Table 2). Regarding the 11 patients treated with adjuvant radiotherapy, five of them (45.5%) experienced local recurrence, and among the 73 who were not given adjuvant radiotherapy, 15 (20.5%) had local recurrence. In terms of distant metastasis, univariate and multivariate analysis both identified larger size as an independent risk factor for unfavorable distant metastasis (HR 1.09 [95% CI: 1.03–1.15], *P* = 0.003) (Table 3). A history of previous surgery and the administration of adjuvant chemotherapy were independent risk factors for favorable distant metastasis (HR 0.13 [95% CI: 0.02–0.95]; *P* = 0.044) (HR 0.39 [95% CI: 0.18–0.83]; *P* = 0.014) (Table 3). For DSS, univariate and multivariate analyses revealed that larger size and no surgery were independent risk factors for unfavorable DSS (HR 1.13 [95% CI: 1.06–1.19]; *P* < 0.001; HR 3.87 [95% CI: 1.61–9.28]; *P* = 0.002 respectively) (Table 4). Previous surgery and adjuvant chemotherapy were independent risk factors for favorable DSS (HR 0.19 [95% CI: 0.04–0.84]; *P* = 0.028; HR 0.36 [95% CI: 0.16–0.77]; *P* = 0.009 respectively) (Table 4).

**Table 2:** Cox regression analysis of local recurrence-free survival in the patients who had localized dedifferentiated chondrosarcoma and received surgery.

Univariate analysis			
Variable (*n* = 84)	No. of patients	Hazard ratio (95% CI)	*P* value
Age (years)	84	0.99 (0.96–1.02)	0.554
Sex			
Male	49	1	
Female	35	1.35 (0.56–3.25)	0.499
Previous surgery, *n* (%)			
No	79	1	
Yes	5	1.95 (0.45–8.43)	0.371
Site			
Trunk	31	1	
Upper limb	17	0.30 (0.06–1.35)	0.117
Lower limb	36	0.58 (0.23–1.47)	0.247
Tumor size (cm)	84	0.95 (0.85–1.05)	0.338
Surgical margin			
R0	70	1	
R1 or R2	14	3.39 (1.35–8.52)	0.010*
Adjuvant radiotherapy			
No	73	1	
Yes	11	2.41 (0.87–6.64)	0.090
Adjuvant chemotherapy			
No	67	1	
Yes	17	0.75 (0.25–2.24)	0.605
Multivariate analysis			
Variable	No. of patients	Hazard ratio (95% CI)	*P* value
Surgical margin			
R0	70	1	
R1 or R2	14	3.39 (1.35–8.52)	0.010*

* Statistically significant.

**Table 3:** Cox regression analysis of distant metastasis-free survival in patients with localized dedifferentiated chondrosarcoma.

Univariate analysis			
Variable (*n* = 98)	No. of patients	Hazard ratio (95% CI)	*P* value
Age (years)	98	1.01 (0.99–1.03)	0.502
Sex			
Male	61	1	
Female	37	0.99 (0.57–1.71)	0.977
Previous surgery			
No	91	1	
Yes	7	0.20 (0.03–1.44)	0.110
Site			
Trunk	41	1	
Upper limb	20	0.71 (0.30–1.70)	0.441
Lower limb	37	1.81 (1.00–3.29)	0.049*
Tumor size (cm)	98	1.06 (1.00–1.12)	0.049*
Surgical margin			
R0	70	1	
R1 or R2	14	0.77 (0.34–1.72)	0.519
No surgery	14	1.25 (0.53–2.95)	0.614
Adjuvant radiotherapy			
No	87	1	
Yes	11	1.92 (0.93–3.94)	0.076
Adjuvant chemotherapy			
No	78	1	
Yes	20	0.54 (0.27–1.12)	0.097
Multivariate analysis			
Variable	No. of patients	Hazard ratio (95% CI)	*P* value
Previous surgery			
No	91	1	
Yes	7	0.13 (0.02–0.95)	0.044*
Tumor size (cm)	98	1.09 (1.03–1.15)	0.003*
Adjuvant chemotherapy			
No	78	1	
Yes	20	0.39 (0.18–0.83)	0.014*

* Statistically significant.

**Table 4:** Cox regression analysis of disease-specific survival in patients with localized dedifferentiated chondrosarcoma.

Univariate analysis			
Variable (*n* = 98)	No. of patients	Hazard ratio (95% CI)	*P* value
Age (years)	98	1.02 (0.99–1.04)	0.147
Sex			
Male	61	1	
Female	37	0.86 (0.48–1.53)	0.609
Previous surgery, *n* (%)			
No	91	1	
Yes	7	0.45 (0.11–1.86)	0.273
Site			
Trunk	41	1	
Upper limb	20	0.67 (0.30–1.53)	0.345
Lower limb	37	1.07 (0.58–1.97)	0.836
Tumor size (cm)	98	1.08 (1.02–1.15)	0.009*
Surgical margin			
R0	70	1	
R1 or R2	14	1.33 (0.64–2.78)	0.450
No surgery	14	2.08 (0.91–4.72)	0.081
Adjuvant radiotherapy			
No	87	1	
Yes	11	1.38 (0.62–3.07)	0.435
Adjuvant chemotherapy			
No	78	1	
Yes	20	0.55 (0.26–1.17)	0.123
Multivariate analysis			
Variable	No. of patients	Hazard ratio (95% CI)	*P* value
Previous surgery, *n* (%)			
No	91	1	
Yes	7	0.19 (0.04–0.84)	0.028*
Tumor size (cm)	98	1.13 (1.06–1.19)	<0.001*
Surgical margin			
R0	70	1	
R1 or R2	14	1.74 (0.81–3.73)	0.158
No surgery	14	3.87 (1.61–9.28)	0.002*
Adjuvant chemotherapy			
No	78	1	
Yes	20	0.36 (0.16–0.77)	0.009*

* Statistically significant.

## Discussion

Our analysis revealed that distant metastases at diagnosis significantly shortened survival, consistent with previous reports [4, 7, 8, 11, 12]. In localized DDCS, inadequate surgical margins were a key risk factor linked with local recurrence, but we found only a limited effect of adjuvant radiotherapy in exerting local control. Patients who underwent initial surgery at a previous hospital and patients with small tumor size had a lower risk of distant metastases, and adjuvant chemotherapy decreased the risk of distant metastases. Survival was longer in patients who underwent initial surgery at a previous hospital and in patients with smaller tumor sizes, and adjuvant chemotherapy improved survival.

Prognostic factors for improved survival in DDCS have been studied repeatedly, and male sex [12], older age [6, 11, 12], location in the trunk [6], larger-sized tumors [7, 8], extraosseous extension [7], pathological fracture [6, 7], metastasis at diagnosis [4, 7, 8, 11, 12], no surgery of primary site [6], positive surgical margins [6], poor performance status [20], radiotherapy [12], histology of undifferentiated pleomorphic sarcoma [4, 7], high proportion of dedifferentiated components [4], and high levels of C-reactive protein [10] have all been described as prognostic factors indicating poor survival in DDCS.

Kawaguchi et al. performed a multivariate analysis of the outcomes of 34 patients diagnosed with localized DDCS and reported that survival was significantly improved by ifosfamide-based chemotherapy (HR, 0.2; 95% CI, 0.09–0.6; *P* = 0.003) [9]. Miao et al. also reported improved survival with adjuvant chemotherapy when they performed a multivariate analysis that included 72 DDCS patients (HR: 0.23, 95% CI: 0.12–0.44, *P* < 0.001) [7]. Chemotherapy regimens effective against the dedifferentiated component of DDCS (osteosarcoma or undifferentiated pleomorphic sarcoma) were selected [7, 9]. For osteosarcoma of the extremities, a randomized controlled trial (RCT) has demonstrated the effectiveness of adjuvant chemotherapy in improving survival [21]. On the other hand, several studies have reported poor prognostic benefit of adjuvant chemotherapy, including studies by Grimer et al., who published a report concluding that adjuvant chemotherapy did not show an association with improved survival in 242 localized DDCS patients (HR 1.32 [95% CI 0.98 to 1.83]; *P* = 0.07) [6]. A recent systematic review found that in localized cases of DDCS with no distant metastases at diagnosis, 5-year survival rates showed little difference between patients treated with surgery plus adjuvant chemotherapy and those receiving surgery alone [22]. The 5-year survival rate for the former group was 28.2% (51/181 patients), while it was 24.0% (90/375 patients) for the latter [22]. Overall, the pooled odds ratio was found to be 1.25 (95% CI, 0.80–1.94; *P* = 0.324) [22]. However, when only localized peripheral-type DDCS was included, adjuvant chemotherapy showed an association with extended survival time (*P* = 0.03) [5].

Grimer et al. reported no benefit from adjuvant radiotherapy in 242 patients with DDCS who underwent surgery [6]. Furthermore, Lee et al. stated that in 227 chondrosarcoma patients, including 20 with DDCS, 56 received adjuvant radiotherapy, but this did not improve prognosis [23]. Chondrosarcomas are particularly resistant to conventional radiotherapy, which is more effective against cells that are rapidly dividing due to the dense extracellular matrix of cartilage and the presence within the tumor tissue of some cells that may grow slowly [24]. In fact, it is necessary to deliver doses of over 70 Gy to achieve local control. However, due to the surrounding nerves and intestinal tract, it can be difficult to reach this dose with conventional radiotherapy, and the tolerated dose is far below 70 Gy [25]. Therefore, in the case of DDCS, radiotherapy with a dose of more than 70 Gy is required after R1/R2 resection using special techniques such as particle beams [14].

Grimer et al. also reported that among 254 surgically-treated DDCS patients, local recurrence was associated with surgical margins. Local recurrence was documented in 11% of patients who received appropriate resection (R0) but 29% in those who received inappropriate resection (R1 or R2) (*P* = 0.0016) [6].

In an analysis of 159 patients with DDCS from the SEER database, tumor diameter > 8 cm (T2 lesion) was found to show a significant association with increased mortality (HR 1.74, 95% CI: 1.01–3.00, *P* = 0.046) [8]. The reason why the rates of both distant metastasis and mortality were lower in patients who had undergone initial surgery at a previous hospital may be that the rate of growth was so slow that the previous doctor mistakenly thought the tumor was benign [26]. Grimer et al. reported that, for patients with localized DDCS, survival rates were significantly lower in patients who had not been treated by surgery at the primary site [6].

This study has a number of limitations. First, because it is a retrospective study, there is a bias toward adjuvant radiotherapy and chemotherapy. Adjuvant chemotherapy was frequently given to younger patients. The second aim, because the number of patients and events was too small to perform multivariate analysis, was to carry out a stepwise multivariate analysis including all variables. Therefore, researchers should be circumspect when interpreting these results. Third, our data do not include histologic response assessment of neoadjuvant chemotherapy. The percentage of patients in whom the entire specimen showed > 90% necrosis in histologic response assessment of neo-adjuvant chemotherapy for DDCS has been reported to be 0–33% [4, 6, 13]. In conventional osteosarcoma, necrosis of 90% or more of the entire specimen evaluated histologically after neo-adjuvant chemotherapy is reported to reach 46% [27], but this is lower in DDCS. Fourth, our data do not include information on radiographic subtypes (central or peripheral). To date, only two studies have reported prognosis according to different radiographic subtypes [4, 5]. Adjuvant chemotherapy showed no association with longer survival for the localized and central type of DDCS (*P* = 0.88) [4] but did show a link with extension of survival for the localized and peripheral-type DDCS (*P* = 0.03) [5]. Thus, patients included in this study may have a higher proportion of peripheral-type disease. Fifth, there are no data on pathological fractures. Pathological fractures may result in local seeding of tumor cells via hematoma, making it difficult to achieve tumor resection at the negative margin and reducing survival [28]. Conversely, Sambri and colleagues stated in their study that pathological fractures had no significant effect on survival [11].

RCTs can avoid many of these biases by randomly allocating participants into groups. However, conducting RCTs for rare cancers like DDCS is extremely difficult. In the future, it will be necessary to retrospectively register detailed information on patients with DDCS and conduct a matched comparative study with a single-arm prospective study [13] lacking a control group in which 57 patients with DDCS undergo surgery and adjuvant chemotherapy to confirm the effects of adjuvant chemotherapy on localized DDCS.

## Conclusions

The patients diagnosed with DDCS with distant metastases had a poor prognosis. In localized DDCS, adjuvant radiotherapy showed limited efficacy in local control, while adjuvant chemotherapy was associated with improved survival outcomes. Further research is needed to optimize treatment protocols.

## Data Availability

The datasets generated and/or analyzed during the current study are not publicly available due to privacy considerations but are available from the corresponding author upon reasonable request.
